# Uncoupling proteins, dietary fat and the metabolic syndrome

**DOI:** 10.1186/1743-7075-3-38

**Published:** 2006-09-12

**Authors:** Janis S Fisler, Craig H Warden

**Affiliations:** 1 Department of Nutrition, University of California, Davis, CA 95616 USA; 2 Rowe Program in Genetics, Department of Pediatrics, Division of Clinical Nutrition, Endocrinology and Vascular Biology, and Section of Neurobiology, Physiology, and Behavior, University of California, Davis, CA 95616 USA

## Abstract

There has been intense interest in defining the functions of UCP2 and UCP3 during the nine years since the cloning of these UCP1 homologues. Current data suggest that both UCP2 and UCP3 proteins share some features with UCP1, such as the ability to reduce mitochondrial membrane potential, but they also have distinctly different physiological roles. Human genetic studies consistently demonstrate the effect of UCP2 alleles on type-2 diabetes. Less clear is whether UCP2 alleles influence body weight or body mass index (BMI) with many studies showing a positive effect while others do not. There is strong evidence that both UCP2 and UCP3 protect against mitochondrial oxidative damage by reducing the production of reactive oxygen species. The evidence that UCP2 protein is a negative regulator of insulin secretion by pancreatic β-cells is also strong: increased UCP2 decreases glucose stimulated insulin secretion ultimately leading to β-cell dysfunction. UCP2 is also neuroprotective, reducing oxidative stress in neurons. UCP3 may also transport fatty acids out of mitochondria thereby protecting the mitochondria from fatty acid anions or peroxides. Current data suggest that UCP2 plays a role in the metabolic syndrome through down-regulation of insulin secretion and development of type-2 diabetes. However, UCP2 may protect against atherosclerosis through reduction of oxidative stress and both UCP2 and UCP3 may protect against obesity. Thus, these UCP1 homologues may both contribute to and protect from the markers of the metabolic syndrome.

## Background

The uncoupling proteins 1, 2 and 3 (UCP1, UCP2, and UCP3) are members of the super family of anion carrier proteins located in the inner membrane of mitochondria. UCP1 is found only in brown fat mitochondria of mammals. Studies, beginning in the 1960s, identified the function of UCP1 in providing heat and decreasing energy efficiency through dissipation of the proton electrochemical gradient across the inner mitochondrial membrane of brown adipose tissue without the generation of ATP (reviewed in [1]). Thus, the function of UCP1 was known before the gene was cloned. On the other hand, UCP2 [2] and UCP3 [3,4], were identified in 1997 by reverse cloning, i.e. by 'mining' databases of expressed sequence tags or from similarity to UCP1 in cDNA libraries (reviewed in [5]). UCP2 and UCP3 have 59% and 57% identity, respectively, with UCP1, and 73% identity with each other [6].

Since their cloning in 1997 through July 2006, 945 publications in English, of which 131 are reviews, involving UCP2 and/or UCP3 are listed in the PubMed database [7], indicating the interest in and the uncertainty regarding the functions of these UCP1 homologues. This brief review will discuss the tissue distribution, reactions, and physiological functions of UCP2 and UCP3. Finally, regulation of these UCPs by dietary fat and the relevance of the UCP proteins to the metabolic syndrome will be discussed. For greater detail on these topics the reader is referred to the following recent reviews [5,6,8-11].

## Tissue distribution

UCP2 mRNA is widely distributed with greatest amounts found in spleen, thymus, pancreatic β-cells, heart, lung, white and brown adipose tissue, stomach, testis, and macrophages, and lesser amounts found in brain, kidney, liver, and muscle (reviewed in [5,10]). Although UCP2 mRNA is found in most organs of the body, there are two difficulties in the determination of which tissues translate UCP2 mRNA into UCP2 protein. First, since UCP2 mRNA is expressed in macrophages, including resident macrophages in several tissues such as brain, liver and lung (reviewed in [10]), it is difficult to determine if the UCP2 protein is in the macrophages or in the parenchymal cells of the tissue. Second, many early studies used antibodies to UCP2 protein whose specificity was not verified in UCP2 knockout models. Such verification is essential since most "UCP2" antibodies bind to proteins of the same molecular weight as UCP2 even in knockout homozygotes. A few authors have hypothesized, based on Western blots, that UCP2 protein is much less widely distributed than is UCP2 mRNA. However, presence or abundance of UCP2 protein have not been assessed by more sensitive methods. UCP2 protein is clearly found in parenchymal cells of brain, spleen, white adipose tissue and β-cells of the pancreas, and in macrophages in brain, liver and lung but it must be remembered that the amount of UCP2 mRNA may not necessarily predict the amount of UCP2 protein in any tissue or under varying conditions [12].

Both UCP3 mRNA and protein, on the other hand, are found only in skeletal muscle and in heart. UCP3 mRNA is also found in brown fat, but whether or not the protein has been identified there is controversial.

## Reactions

The primary function of UCP1 is to allow a leak of protons through the inner mitochondrial membrane of brown fat thereby uncoupling substrate oxidation from phosphorylation of ADP to ATP resulting in rapid oxygen consumption, heat production, and energy wastage. This function of UCP1 is mediated by the sympathetic nervous system and norepinephrine in brown adipose tissue and is stimulated by fatty acids and inhibited by purine nucleotides. When UCP2 and UCP3 were first cloned, it was speculated that these proteins would fulfill much the same function, albeit in tissues other than brown fat. However, it quickly became evident that the UCP homologues were not thermogenic to the same degree as UCP1 [13]. Expression of UCP2 and UCP3 can be as much as 1000 fold less than expression of UCP1 [12] and therefore, the expected rate of proton conductance due to UCP2 or UCP3 would be much lower than that produced by UCP1.

Two mechanisms have been proposed to explain the reactions catalyzed by UCP1-3; net proton transport across the membrane and/or export of fatty acid anions (reviewed in [6,9]) both of which are generally accepted for UCP1. UCP2 and UCP3 do, in fact, increase net proton conductance across the mitochondrial inner membrane, but only when activated either directly or indirectly by superoxide or by derivatives of reactive oxygen species; neither UCP2 nor UCP3 appear to catalyze basal proton conductance. Activation, in turn, requires fatty acids (reviewed in [9]). Both UCP2 and UCP3, like UCP1, are inhibited by purine nucleotides.

## Physiological functions

These novel uncoupling proteins have several hypothesized functions including thermogenesis in certain tissues, protection from reactive oxygen species (ROS), mediation of insulin secretion, neuroprotection and export of fatty acids. Several of these functions are related to the etiology of the metabolic syndrome.

### Thermogenesis

Energy wastage and protection from obesity were initially suggested to be significant functions of UCP2 and UCP3 due to their homology to UCP1 and their distribution in tissues, such as white adipose tissue and muscle, that could dissipate energy. Gene expression of UCP2 and UCP3 increases during fasting [14], opposite of what would be expected for a thermogenic compound, and neither UCP2 nor UCP3 knockout mice are obese [15,16], providing evidence against these proteins contributing to whole body thermogenesis. Transgenic mice over-expressing a UCP2/UCP3 construct, however, are leaner than wildtype [17,18] and those overexpressing only UCP3 in skeletal muscle are leaner despite hyperphagia [19]. However, there is a concern that uncoupling in over-expression studies is an artifact and does not reflect the native function of the protein in the cell. Thus, caution is appropriate in ascribing a whole body thermogenic function and protection against obesity to UCP2 or UCP3 (reviewed in [8,9]).

Two lines of evidence suggest that, under specific conditions, the UCP homologues can have a thermogenic function. Higher temperature is co-localized with UCP2 mRNA within certain areas of the brain suggesting that UCP2 could function as a thermogenic protein in the microenvironment of the brain [20,21]. In UCP3 knockout mice, the thermogenic response to the drug MDMA (ecstasy) is significantly reduced, suggesting that UCP3 in muscle can affect whole body thermogenesis in this non-physiological condition [22].

### Protection from oxidative damage

**Figure 1:**
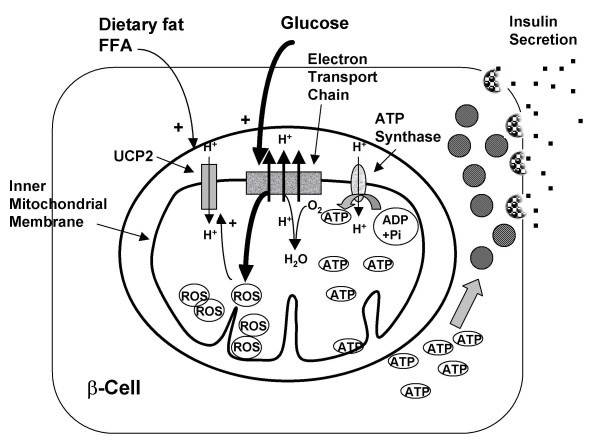
**Insulin secretion during high glucose usage**. (figure adapted from [6]) In the normal fed state UCP2 expression is low. Metabolism is shifted toward glucose oxidation resulting in ROS production and generation of ATP. Insulin secretion is stimulated by the resulting high ATP/ADP ratio. Elevated ROS levels feed back to increase UCP2 thereby mildly uncoupling respiration, reducing membrane potential and ROS production.

**Figure 2:**
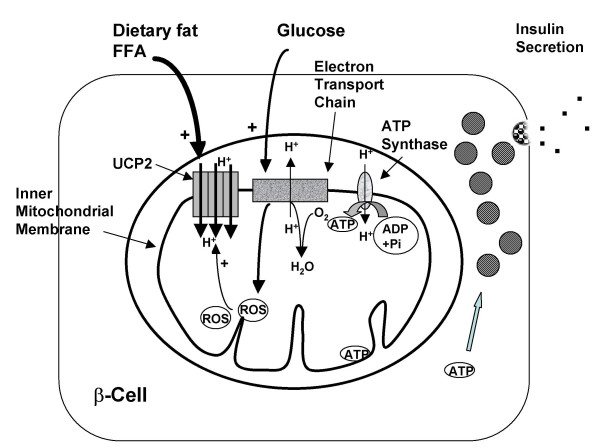
**Insulin secretion during high fatty acid usage**. (figure adapted from [6]) When FFA are elevated, such as with a high fat intake or during fasting, UCP2 expression is increased, the mitochondrial membrane potential is reduced, and fewer electrons pass through the electron transport chain resulting in reduced ATP production. The lower ATP/ADP ratio results in diminished insulin secretion.

ROS production occurs to a large extent in mitochondria and is very sensitive to the mitochondrial membrane potential. When electron flow through the respiratory chain is elevated, 'backed up' electrons may react with oxygen to produce ROS (Figure 1). It is proposed that one function of both UCP2 and UCP3 is to mildly uncouple respiration, allowing a more rapid electron flux, thus reducing membrane potential resulting in reduced ROS production (Figure 2). Superoxides and derivatives of ROS are known to activate GDP-sensitive proton conductance catalyzed by UCP2 or UCP3, thus forming a feedback loop for control of ROS production. Since even mild uncoupling has a large effect on reducing ROS production, this hypothesis has strong support and is now generally accepted (reviewed in [6,8,10]. UCP2 knockout mice have elevated ROS production in macrophages [16] and pancreatic islet cells [23] and UCP3 knockout mice have elevated ROS in muscle [24] further supporting the role of these UCPs in protecting against ROS production and tissue oxidative damage.

### Mediation of insulin secretion

It is now well established that UCP2 plays an important role in regulation of insulin secretion. Pancreatic β-cells secrete insulin in response to a meal by sensing the ATP/ADP ratio resulting from glucose metabolism in the cell (Figure 1). UCP2, by mildly increasing proton leak, decreases the ATP/ADP ratio of the cell thus reducing the effect of glucose on insulin secretion (Figure 2) (reviewed in [6,8,10]). Pancreatic islets from UCP2 knockout mice (UCP2^-/-^) have increased insulin secretion in response to glucose and these mice have higher blood insulin and lower blood glucose than wildtype supporting the role of UCP2 as a negative regulator of insulin secretion in the whole animal [25]. Double mutant Lep^ob/ob ^UCP2^-/- ^mice have improved β-cell function independent of obesity [25].

### Neuroprotection

A number of studies suggest that UCP2 functions in neuroprotection, including following cerebral ischemia or traumatic injury and prevention of seizures or Parkinson's disease (reviewed in [10]). UCP2 is found in the inner mitochondrial membranes in several brain regions and is often co-expressed with neuropeptides in both rodents and primates [21,26]. Several mechanisms contributing to neuronal cell death, including excitotoxicity, mitochondria-mediated cell death and ROS damage are affected by UCP2 (reviewed in [10]). Exposing the neurons to periods of sub-lethal ischemia preconditions the cells to survive ischemic insults that would normally be lethal. This preconditioning is protein synthesis dependent. UCP2 is upregulated in the hippocampus following ischemic preconditioning [27], suggesting that UCP2 functions as part of the response to ischemic and oxidative stress in the neurons. Since ROS production and oxidative damage are involved in most neurodegenerative disorders, UCP2 induction was proposed to have a potential therapeutic effect in the treatment of epilepsy, Parkinson's disease, Alzheimer's disease, brain hypoxia and stroke [10]. UCP2, in fact, was shown to have a neuroprotective effect in a mouse model of Parkinson's disease [28]. Opposed to increased UCP2 being neuroprotective is a study showing that UCP2 knockout mice were less sensitive to ischemia following cerebral artery occlusion than wildtype mice [29]. These authors propose that UCP2 is not directly involved in the regulation of ROS production, but rather is acting through regulation of mitochondrial glutathione [29].

### Export of fatty acids

UCP3 has been proposed as a transporter of fatty acid anions out of mitochondria [30] by analogy with UCP1. The fatty acid cycling model, proposed for UCP1 in 1996 [31], suggests that UCP3, by transporting fatty acids out of mitochondria, protects the mitochondria from the toxic effects of fatty acid anions or peroxides [11]. The hypothesis is attractive in that it is consistent with observations that UCP3 expression is correlated with improved fatty acid oxidation when fatty acid supplies are high, for example with fasting or a high fat diet (reviewed in [6,8,11]). Also, muscle UCP3 protein levels are increased when rats are fed a diet high in long chain triglycerides but not a diet high in medium chain triglycerides which are oxidized via a different pathway [32], again linking fatty acid oxidation with UCP3. Results from UCP3 knockout mice are not consistent, however, some showing reduced rates of fatty acid oxidation and others no effect (reviewed in [6,8]. Thus the proposal that UCP3 protects against fatty acid toxicity in the mitochondria remains to be confirmed.

## Effect of dietary fat

UCP2 and UCP3 expression is elevated during fasting [33] or other states, including feeding of high fat diets, where circulating fatty acid levels are elevated and there is a shift from carbohydrate to lipid oxidation (Figures 1 and 2). Most animal models show up-regulation of UCP2 and/or UCP3 by high fat diets [2,34-37], although this has not been universally observed. Up-regulation of UCP expression depends on strain and tissue type: a high fat diet increased UCP3 mRNA expression in skeletal muscle of C57BL/6J mice [37] and rats [38] but increased only slightly UCP2 expression in white adipose tissue of AKR mice and not at all in C57BL/6J mice [37] or in rats [38]. The up-regulation by a high fat diet of UCP3 protein also occurs in human skeletal muscle [39].

A high fat, ketogenic diet increases UCP2 mRNA and protein levels and reduces ROS production in the brain [35,40]. Conversely, substitution of a low fat diet to immature rats reduces UCP2 levels and increases ROS production and seizure-induced excitotoxicity [41]. Thus, the ketogenic diet may be neuroprotective by diminishing ROS production through activation of UCP2 in the brain [35]. Indeed, UCP2 up-regulation in the brain is proposed as the mechanism by which a ketogenic diet reduces pediatric seizures.

## Relevance to the metabolic syndrome

Features of the metabolic syndrome include central adiposity, increased plasma triglycerides and free fatty acids, insulin resistance, hyperglycemia, increased inflammation and hypertension, leading to increased risk of type-2 diabetes, atherosclerosis and stroke. Several of these features are influenced by UCP2 and/or UCP3.

Genetic association of natural polymorphisms with phenotypes in humans provides an independent method to determine the *in vivo *functions of UCP2 and UCP3. Thus, one can demonstrate the influence of UCP2 on type-2 diabetes or BMI in an association study even without proving the underlying biochemical reaction carried out by these proteins. Indeed, genetic studies can be used to guide biochemical studies towards understanding the biochemistry of UCP2 and UCP3.

### Obesity

A review of human genetic studies examining expression of UCP2 or UCP3 and the propensity to obesity suggested that some obesity related phenotypes are significantly associated with these UCPs. A UCP2 insertion/deletion variant was associated with BMI in 4 studies and polymorphisms of UCP3 were associated with BMI in 2 studies (reviewed in [42]). These authors concluded that since the UCP2 insertion/deletion variant association with BMI was observed in a variety of ethnic groups, the variant itself underlies the association with BMI [42]. Population studies showed that a common polymorphism in the UCP2 promoter, -866G/A, was associated with a reduced risk of obesity in Caucasian Europeans [43,44]. However, the data suggesting an effect of UCPs on obesity remains uncertain, with two recent papers reporting no linkage or association with UCP2 or UCP3 alleles [45,46], while another reports association of UCP3 alleles with measures of body composition in women [47].

### Type-2 diabetes

As noted above, UCP2 is a regulator of insulin secretion and it is proposed that increased expression of UCP2 in pancreatic β-cells results in chronic down-regulation of glucose stimulated insulin secretion (Figure 2) leading to β-cell dysfunction and the development of type-2 diabetes (reviewed in [6,8,10]). Several population studies now show that a common functional polymorphism in the UCP2 promoter (-866G/A, the same polymorphism found to be associated with resistance to obesity in Caucasians) enhances UCP2 transcriptional activity [43] and increases the risk of developing type-2 diabetes [44]. This polymorphism is associated with impaired β-cell function [48], impaired insulin sensitivity [49], and with earlier, more severe diabetes [50].

Interestingly, the same UCP2 -866G/A polymorphism and a -55C/T polymorphism in UCP3 are both associated with significantly reduced prevalence of diabetic neuropathy in type-1 diabetics [51]. Presumably these polymorphisms prevent mitochondrial mediated neuronal injury and thus protect against diabetic neuropathy. Another common polymorphism in UCP2, -55A/V, was examined in the Coronary Artery Risk Development in Young Adults (CARDIA) study. The -55V/V genotype was positively related to diabetes, perhaps through insulin resistance in individuals with impaired glucose homeostasis [52]. Insulin resistance of type-2 diabetes and obesity involves muscle, liver and adipocytes. UCP2 knockout mice show increased insulin sensitivity and are protected against dietary fat induced insulin resistance [53]. Conversely, data from *in vitro *studies in L6 muscle cells suggest that UCP3 functions to facilitate fatty acid oxidation and minimize mitochondrial ROS production, perhaps thereby reducing muscle insulin resistance [54].

The data presently available suggest that UCP2 up-regulation has opposing effects on different components of type-2 diabetes. Increased UCP2 results in β-cell dysfunction, impaired insulin sensitivity, and earlier, more severe diabetes, but may protect from diabetic neuropathy. Thus, UCP2 is proposed as a diabetes gene [55]. Increased UCP3 may, on the other hand, reduce muscle insulin resistance.

### Atherosclerosis

UCP2 protects against atherosclerosis in animal models [56] potentially through inhibition of ROS production in endothelial cells [57] and inhibition of monocyte accumulation in the artery wall [58]. A common variant in the UCP2 gene is associated with cardiovascular risk in healthy men and with oxidative stress in diabetic men [59].

## Competing interests

The author(s) declare that they have no competing interests.
